# Game-Based Intervention for Adolescent Pregnancy Prevention: Protocol for a Multisite Randomized Controlled Trial Evaluation

**DOI:** 10.2196/88047

**Published:** 2026-10-08

**Authors:** Brittany Rosen, Sydney Hineline, Andrea Meisman, Bin Zhang, Caitlin Holden, Christi Esquivel, Whitney Garney, Kelly Wilson

**Affiliations:** 1 Division of Adolescent and Young Adult Medicine Cincinnati Children's Hospital Medical Center Cincinnati, OH United States; 2 College of Medicine University of Cincinnati Cincinnati, OH United States; 3 Cincinnati Children's Hospital Medical Center Cincinnati, OH United States; 4 The Division of Biostatistics and Epidemiology Cincinnati Children's Hospital Medical Center Cincinnati, OH United States; 5 Texas A&M University College Station, TX United States

**Keywords:** adolescent pregnancy, evaluation study, randomized controlled trial, sexual behavior, social cognitive theory, trial protocol

## Abstract

**Background:**

Existing adolescent pregnancy prevention programs have demonstrated modest effects. Evaluation is needed for interventions aimed at improving intermediary outcomes linked to behaviors associated with adolescent pregnancy prevention, including intentions, communication skills, decision-making skills, access to information, and supportive adult relationships. These intermediary outcomes often serve as mechanisms that decrease sexual behaviors associated with unintended pregnancy and sexually transmitted infection risk and enhance understanding of how interventions influence behavior. The US federal government announced a funding cycle aimed at assessing innovative programs for underserved youth. As 1 of the 12 grantees, we will evaluate Using the Connect (UTC), a novel game-based learning intervention offering opportunities to improve participants’ intention to delay sex, skills related to communication, decision-making, and accessing information, and strengthen their connection to a trusted adult.

**Objective:**

This study describes the clustered randomized controlled trial protocol to assess the effectiveness of UTC on middle school youth’s intention to delay sex, individual-level skills (communication, decision-making, and accessing information), and perception of connection to a trusted adult.

**Methods:**

This multisite trial had planned enrollment for 1150 youth aged 10-14 years from 46 community-based organizations in Texas, Alabama, and Illinois. Sites will be randomized to either UTC or business-as-usual control consisting of routine youth programming (eg, physical activity, career education, and mental health awareness). UTC consists of 4 interactive games, 1 wrap-up activity, and 1 take-home activity focused on communication, decision-making, accessing sexual health information and resources, and strengthening connections with trusted adults. Participants will complete electronic surveys at baseline, immediate post-survey, 3-month follow-up, and 9-month follow-up. Outcomes will be assessed using adapted survey measures of intention to delay sexual intercourse, communication skills, decision-making skills, skills to access sexual health information and resources, and perceived connection to trusted adults. To decrease attrition, we will use an evidence-based retention model. We will conduct sensitivity analyses to assess the robustness of findings.

**Results:**

Funding was received in September 2021 through the Personal Responsibility Education Innovative Strategies funding opportunity within the Personal Responsibility Education Program of the Department of Health and Human Services. Site recruitment and enrollment began in June 2023. Between June 15, 2023, and May 31, 2025, 1338 youth have been enrolled in the study. Follow-up data collection was completed in April 2026. As of July 2026, all study activities have concluded, and data from 1231 participants were included in the final analysis, which is being completed. The final evaluation report will be submitted by September 2026. Findings will be disseminated through scientific conferences and peer-reviewed publications.

**Conclusions:**

This study will evaluate and contribute evidence regarding a novel, game-based intervention to strengthen protective factors associated with adolescent pregnancy prevention in middle school–aged youth.

**Trial Registration:**

ClinicalTrials.gov NCT05927194; https://clinicaltrials.gov/study/NCT05927194

**International Registered Report Identifier (IRRID):**

DERR1-10.2196/88047

## Introduction

### Overview

For over 30 years, the US adolescent birth rate has been declining [1]. However, the United States ranks highest on the list of industrialized nations for rates of adolescent pregnancy and teen births [1-3]. Adolescent pregnancy and teen births are linked to adverse economic, health, and educational outcomes for both parents and children [4]. Specifically, teen parents are less likely to continue their education, which leads to a decrease in economic opportunities and a reduction in lifetime earnings, while their children are more likely to experience preterm birth and lower birth weight which is highly associated with adverse developmental and social outcomes [4,5]. Moreover, disparities in teen birth rates persist. The US teen birth rates among American Indian/Alaska Native, Native Hawaiian/Pacific Islander, Black, and Hispanic adolescents were twice the rate compared to White adolescents, with additional disparities occurring across geographic regions [6]. Texas has historically reported adolescent birth rates above the national average. In response, substantial federal and state investments have focused on adolescent pregnancy prevention programming targeted to youth populations at increased risk for unintended pregnancy. The adverse health, developmental, and economic outcomes and persistent disparities underscore the need for innovative, developmentally appropriate, and accessible pregnancy prevention interventions for adolescents.

To address the public health concern of adolescent pregnancy in the United States, most interventions have focused on individual-level factors, such as sexual health knowledge, attitudes, decision-making skills, and behaviors associated with adolescent pregnancy prevention [7-9]. Recent efforts in both the United States and internationally have begun recognizing that sexual health is influenced by interactions between knowledge, skills, social, and environmental factors [10,11]. Thereby, prevention programs are increasingly incorporating parent and community engagement and creating supportive environments [9,12,13]. However, maintaining adolescent engagement throughout such programming remains a challenge. This has increased interest in innovative intervention approaches providing opportunities for active and engaged learning for skill development [14,15].

Game-based learning (GBL) has the potential to be both engaging and educational [16,17]. While traditional games aim to entertain the user, GBL aims to educate the user through gameplay [18]. GBL is an effective strategy that is highly attractive and motivating for participants [19,20]. When designed appropriately, GBL participants are motivated, focused, and engaged to the point of repeated playing or returning to the game over time [19]. GBL cultivates critical thinking, motivates youth to apply knowledge and skills, and increases confidence levels more efficiently than traditional teaching methods [21,22]. According to GBL theoretical underpinnings, games can support education and skill development in communities with limited resources or social norms that discourage discussion of taboo topics, such as sexual health, and when developed collaboratively with community stakeholders, can also address sociocultural norms that may be overlooked in traditional programming [20]. Thus, GBL provides opportunities and situations to practice communication and decision-making skills, receive feedback, and learn about consequences in a safe, low-risk environment.

To develop a GBL intervention, we used the human-centered design (HCD) process to ensure the intervention reflected the needs, preferences, and experiences of the community. As a first step, a nonprofit community organization participated in an HCD “boot camp” led by the Innovative Teen Pregnancy Prevention Programs (iTP3) team [23]. During this boot camp, HCD strategies and brainstorming of ideas led to an innovative adolescent pregnancy prevention program idea for their community. After intensive activities to gain insights, a promising idea for a game emerged. To further develop their idea, the design team engaged in a design sprint completing HCD activities to iteratively develop program components. The design team shared their ideas and progress with youth and other community stakeholders for feedback and completed the design sprint with a prototype of *Using the Connect* (UTC), a novel, interactive game-based intervention consisting of 4 in-person stations and 1 take-home activity. By using skills practice, feedback, peer learning, and guided reflection, UTC aims to strengthen protective factors associated with adolescent pregnancy prevention by developing skills in communication, decision-making, and accessing information, as well as perception of connection with trusted adults. UTC was developed to provide a flexible intervention that can be implemented across varying community settings serving youth. For additional details regarding the intervention development process, see Esquivel et al [24].

The resulting intervention, UTC, was then grounded in the social cognitive theory (SCT), which states that changing a person’s behavior is a result of increasing *intention* by adjusting their *self-efficacy*, as well as their *expectations* and *expectancies* [25,26]. Specifically, our conceptual model centers on *intention* to delay sexual intercourse and to use condoms and contraceptives when sexually active, *self-efficacy* related to communication skills, decision-making skills, and skills to access sexual health information and resources, and *expectations* and *expectancies* related to making decisions for behaviors to prevent pregnancy and sexually transmitted infections (STIs). These SCT constructs were selected due to being identified in empirical studies as salient protective factors against adolescent pregnancy [27]. In addition, we also selected to include youth’s *perception of connection to trusted adults* in our conceptual model, as this construct has been shown to be associated with adolescents’ overall well-being [28].

As a next step, we aligned our conceptual model’s constructs with appropriate behavior change techniques (BCTs). BCTs are defined as intervention components and activities designed to modify the specific behavior or health outcome [29]. Examples of BCTs include feedback, instructions on how to perform behaviors, information about health consequences, and demonstration of the behavior [30]. Our selected BCTs were then operationalized and aligned with the intervention’s games and activities. The games and take-home activity were systematically organized and linked with the corresponding BCTs and conceptual model constructs to guide our rigorous impact evaluation.

UTC was formatively tested in North Carolina and Texas with positive results [24]. Specifically, the youth reported the design of UTC was highly acceptable, the games and content were engaging and relatable, and that they learned knowledge and skills around adolescent development, accessing information, communication, and making decisions related to pregnancy prevention. Youth stated they would recommend the program to friends, would participate in the program again, and use the information they learned in the future [24]. For additional details regarding the usability testing, see Esquivel et al [24].

### Study Purpose

Over the past decade, substantial US federal investments have strengthened the adolescent pregnancy prevention evidence base through the Teen Pregnancy Prevention (TPP), Personal Responsibility Education Program (PREP), and Personal Responsibility Education Program Innovative Strategies (PREIS) initiatives [31]. Collectively, these programs have supported the development, implementation, and rigorous evaluation of interventions designed to reduce adolescent pregnancy and associated sexual risk behaviors among diverse populations of youth. Lessons learned from these federally funded evaluations have highlighted the importance of testing innovative interventions, examining intermediary protective factors that may influence sexual health behaviors, and disseminating detailed evaluation protocols to support transparency, replication, and methodological rigor [32,33]. As a result, there remains a need for rigorous randomized trials that evaluate novel approaches to adolescent pregnancy prevention while clearly documenting intervention theory, implementation procedures, and evaluation methods [33,34].

As 1 of the 12 PREIS grantees evaluating an innovative intervention to decrease adolescent pregnancy prevention, we aim to describe—in detail—the evaluation protocol for a multisite, clustered randomized controlled trial (cRCT). Specifically, we developed a protocol and logic model (Figure 1) to test the impact of UTC on intermediary outcomes. Because most middle school–aged youth have not yet initiated sexual intercourse, behavioral outcomes are expected to demonstrate limited variability during the study period. Therefore, intention to delay sexual intercourse was selected as the primary outcome because it is developmentally appropriate for this population, theoretically aligned with the intervention’s conceptual model, and represents a proximal indicator of future pregnancy prevention behaviors [35]. Furthermore, because UTC centers on promoting skills in communication, decision-making, and accessing information and resources, increasing perception of connection to a trusted adult, and intentions to use condoms and contraception when sexually active, these constructs were included as additional outcomes in the conceptual model and evaluation and are hypothesized to influence intentions and behaviors associated with adolescent pregnancy prevention.

Identifying intermediate outcomes, with potential to serve as protective factors against pregnancy, aligns with the previous calls to action and specifies the first step in how the intervention produces its effect. This, in turn, allows us to apply the results for program improvement and future evaluations. In describing the protocol, we ensure the procedures from this project can be replicated with fidelity to test the intervention in varying settings and populations. Furthermore, this study adds to the methodological foundation to support future grantees in conducting and completing cRCTs. Such information will help overcome the context-dependent success of programs and decrease the resources spent on identifying the factors behind noneffect replication studies.

**Figure 1:**
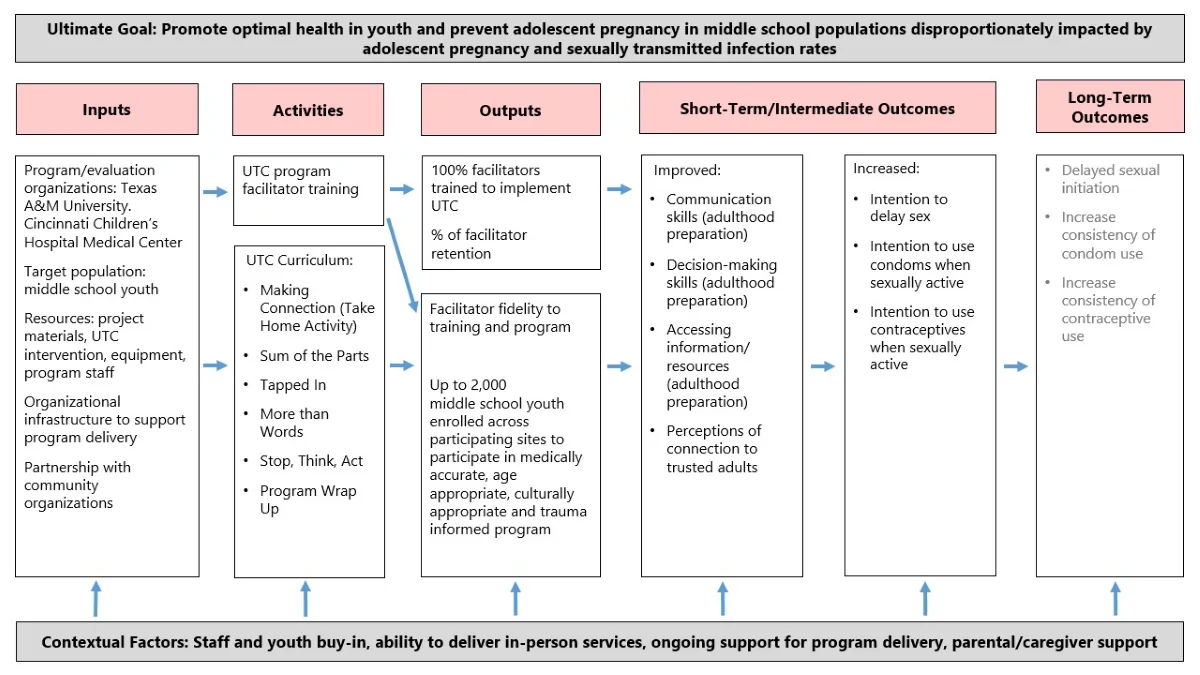
Using the Connect evaluation logic model. UTC: Using the Connect.

Thus, the overall aim of this study is to evaluate the effectiveness of UTC among middle school–aged youth. The objectives are to (1) assess the impact of UTC on youth’s intention to delay sexual intercourse, (2) assess the impact of UTC on communication skills, decision-making skills, and skills to access sexual health information and resources, and (3) assess the impact of UTC on youth’s perceived connection to trusted adults. Given that participation in the intervention is minimal-risk, no specific harms are anticipated; however, adverse events and unintended consequences will be monitored throughout the study. Our study describes the cRCT evaluation protocol to answer the following primary research questions:

1. How does UTC impact middle school–aged youth’s intention to delay sexual intercourse, compared to middle school–aged youth who have not received UTC, at posttest?
2. How does UTC impact middle school–aged youth’s intention to delay sexual intercourse, compared to middle school–aged youth who have not received UTC, at 3-month follow-up?
3. How does UTC impact middle school–aged youth’s communication skills, decision-making skills, skills to access sexual health information and resources, and perception of connection to trusted adults, compared to middle school–aged youth who have not received UTC, at posttest?
4. How does UTC impact middle school–aged youth’s communication skills, decision-making skills, skills to access sexual health information and resources, and perception of connection to trusted adults, compared to middle school–aged youth who have not received UTC, at 3-month follow-up?

## Methods

### Study Design and Registration

This study is a prospective, 2-arm, multisite cRCT with data collection at multiple time points. Study arms include a control arm, in which participants will receive a business-as-usual program, and a treatment arm, in which participants will receive the UTC program. An overview of the trial’s specifications is presented in Table 1.

**Table 1:** Using the Connect trial specifications.

Data category	Information
Registry and trial number	ClinicalTrials.gov: NCT05927194 [36]
Date of registration	June 6, 2023
Financial support	US Department of Health and Human Services, Administration for Children and Families, and Family and Youth Services Bureau
Contact for queries	brittany.rosen@cchmc.org
Title	Using the Connect: game-based intervention (UTC)
Intervention	Active comparator: Using the Connect Passive comparator: business-as-usual
Key inclusion and exclusion criteria	Inclusion: middle school–aged youth (10-14 years of age or 5th-8th grade); ability to complete study activities in English without assistance. Exclusion: unable to read and speak in English.
Study type	Multisite, clustered randomized controlled trial
Date of first enrollment	June 15, 2023
Target sample size	1150 middle school–aged youth
Trial status	Ongoing data collection
Randomization	1:1 block
Analytical framework	Intention-to-treat
Primary outcomes	Youth’s intention to delay sexual intercourse at post and 3-month follow-up; communication skills, decision-making skills, and skills related to accessing sexual health information and resources at post and 3-month follow-up; perception of connection to trusted adults at post and 3-month follow-up
Secondary outcomes	Youth’s intention to delay sexual intercourse at 9-month follow-up; communication skills, decision-making skills, and skills related to accessing sexual health information and resources at 9-month follow-up; perception of connection to trusted adults at 9-month follow-up; intention to use condoms or contraceptives when sexually active at 9-month follow-up

### Study Team

This project will have 2 core functions: UTC implementation and impact evaluation. Thus, this 2-prong approach will use a collaborative model with key leadership comprising of faculty and staff from Texas A&M University (TAMU) serving as the implementation team, and Cincinnati Children’s Hospital Medical Center (CCHMC) serving as the evaluation team. The leads for each team will meet monthly to discuss project status and timeline and address any anticipated challenges. The implementation team (TAMU) will facilitate all implementation activities which include UTC facilitator training, develop site-specific implementation plans, lead monthly conference calls with the full study team, collaborate with facilitators to codevelop the implementation database, provide ongoing technical assistance and support to UTC facilitators, and conduct the implementation evaluation. The evaluation team (CCHMC) will develop and implement all impact evaluation tools and the data collection protocol, provide and oversee data collection training to study sites, assist in collecting and reporting PREIS performance measures data, conduct data management and analyses, and develop and disseminate final evaluation report.

### Setting Overview

We will enroll and randomize 46 community-based organization (CBO) sites located in Texas, Alabama, and Illinois. Using their extensive professional network, the implementation team will identify, recruit, and enroll community-based sites. Strategies to identify sites will include contacting organizations in their professional network serving middle school youth, obtaining recommendations for potential partnering organizations from existing stakeholders, and recruiting at adolescent health conferences. The inclusion criteria for sites include CBOs serving youth aged 10-14 years or youth in 5th to 8th grade. Examples of CBO sites include, but are not limited to, youth development organizations, religious organizations, and after school programs. To participate, eligible sites will (1) be randomized to the treatment or control group, (2) allow youth to complete baseline and immediate follow-up surveys at the site, and (3) allow for study activities, including recruitment, enrollment and consenting procedures, to occur at the site. If the site is eligible, evaluation staff will meet with the site to explain study procedures and expectations. Participating sites will be enrolled in the study. When sites agree to participate, they will complete three steps prior to being randomized: (1) review and sign a memorandum of understanding outlining the study procedures and expectations for recruiting and collecting data from a minimum of 30 youth, (2) participate in study protocol training for recruitment, enrollment, and data collection, and (3) coordinate with the evaluation team to identify dates for youth recruitment, enrollment, and baseline data collection. Once these steps are completed, our randomization procedures will be followed to randomize the site to a study arm. If sites have additional capacity to reach and recruit >30 youth, the implementation team will set a realistic goal in collaboration with the sites to align with project resources and available support.

### Randomization and Blinding

Immediately after enrollment, sites will be randomized in a 1:1 block allocation to either the treatment or control group using a computer-generated randomization schedule to assign a study arm to each site. Given the distinct curriculum differences in programs for each arm, randomization cannot be blinded to the study teams, program facilitators, or participants. However, our statistician will be blinded prior to analysis.

### Site Facilitators and Training

Site facilitators will implement the program and on-site evaluation activities. Eligibility to serve as a site facilitator includes any staff member from a participating CBO or a TAMU implementation staff member. Site facilitators will not be recruited for the study. Instead, participating sites will identify staff members to facilitate UTC as part of their agreement to participate in the study. Site facilitators are not required to hold any particular background or qualifications to be eligible to deliver the intervention, due to the game-based design of the intervention.

The implementation team will train facilitators to successfully implement the UTC program. Implementation training will consist of a 6-hour in-person training course for each site. The training is for any individual at the site who will facilitate UTC. The training will include information on the program’s background as well as provide an opportunity to play the 4 games, the wrap-up activity, and the take-home activity. By having the site facilitators play the games during the training, the implementation team can answer facilitators’ questions as they arise during gameplay. To complete the training, the implementation team will debrief by providing information regarding best practices in the learning environment, ensuring facilitators understand mandatory reporting laws, and allowing time for questions from facilitators.

The evaluation team will train facilitators to successfully complete on-site evaluation responsibilities and activities, including distributing study information sheets to parents, study enrollment, group assent, survey facilitation, ethical research practices, and connecting participants with the study team, as needed. The evaluation training will include a training packet, which requires facilitators to review a site manual introducing the study objectives as well as processes and activities they will be responsible for implementing. Facilitators are expected to use the manual as a reference to the procedures throughout the study duration. After reviewing the manual, the facilitators will complete the required online research training course titled Community Involvement in Research Training (CIRTification). Provided by the University of Cincinnati’s Center for Clinical and Translational Science and Training (CCTST) at no cost, CIRTification is a human research protections training created for community research partners [37]. After completing the CIRTification, facilitators will review 2 training videos. The first training video reviews the study objectives, activities, and responsibilities of the facilitator. The second training video guides the facilitators through using the assent and enrollment scripts provided in the training packet, for conducting the assent and enrollment procedures. During the video, facilitators can pause and practice each component of the assent and enrollment process. Facilitators can rewatch the video at any time during the study. As a last step, facilitators are required to submit regulatory documents, including facilitators’ signed resumes and signed study delegation of authority log, to the evaluation team. After these documents have been submitted, a final meeting between the facilitators and evaluation team will be scheduled to review the training packet and answer any questions. Only after the final meeting will the facilitators receive the final study documents to officially begin recruitment and enrollment.

In addition to the comprehensive training, the evaluation team will use supplemental strategies to support and ensure facilitator success. Strategies include providing facilitators with a 1-page checklist outlining study activities and weekly office hours for facilitators to consult with the evaluation team. The weekly office hours will be a virtual standing meeting. The evaluation team will attend these weekly virtual meetings allowing any facilitators to attend the meeting with emergent questions or concerns.

### Study Population and Inclusion/Exclusion Criteria

All English-speaking youth between the ages of 10 and 14 years (5th-8th grade) receiving services at a participating site during the enrollment period (June 2023-June 2025) are eligible. Because UTC is an English-based program, participants must have the ability to complete the program and surveys in English without assistance.

### Participant Recruitment, Assent, and Enrollment

Facilitators will use methods provided in the training to recruit eligible youth to participate in the program and evaluation activities. During the recruitment and enrollment process, facilitators will provide parents/caregivers with an institutional review board–approved study information sheet. The study information sheet provides contact information for the study team, explains the purpose of the study, the study activities and study payments, as well as any risks involved with survey data collection. Parents/caregivers can withdraw their child from the study at any time by contacting the evaluation team. Youth will use their personal or site provided devices to access and complete the eligibility screening, assent, and contact information form. Facilitators will initiate a group assent process in which eligible participants will be assented together. After completing the eligibility screening form, the assent form will open on youth’s devices. The facilitators will use a script designed by the evaluation team to guide youth through their assent forms. Youth who want to participate in the study will sign and complete the assent form on their device. Facilitators will complete an informed consent process note that will be sent to the evaluation team confirming the group assent procedures were followed. Figure 2 provides a consort diagram of the study flow.

**Figure 2:**
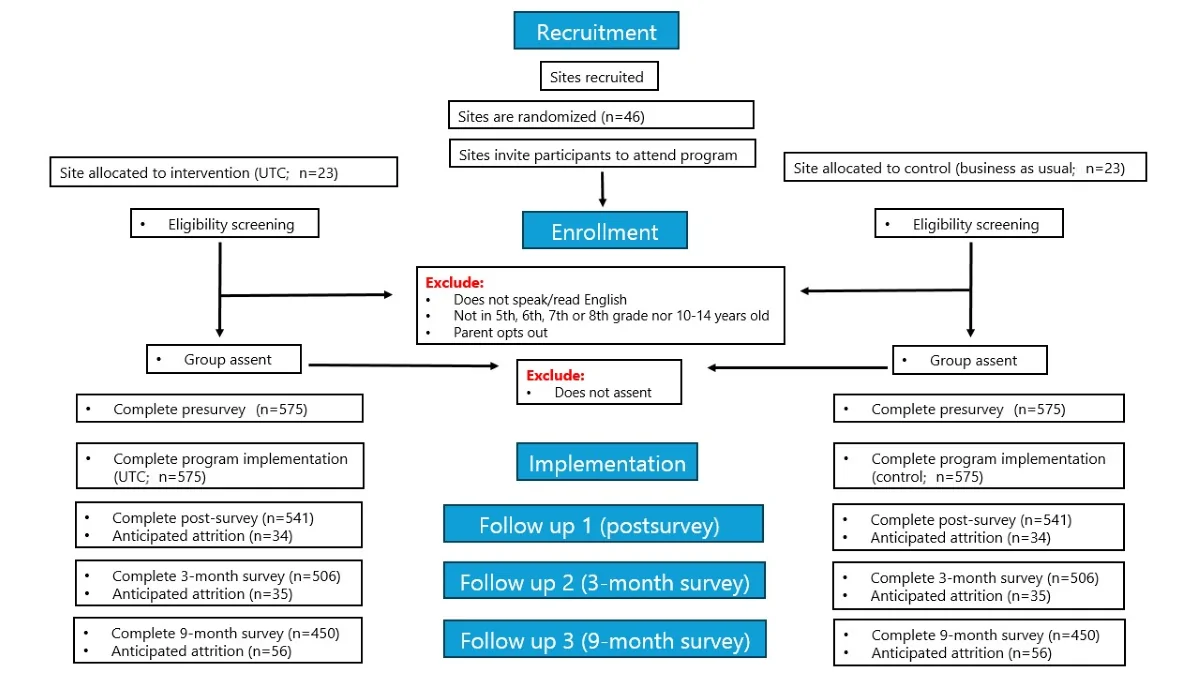
Using the Connect evaluation consort diagram. UTC: Using the Connect.

### Using the Connect

UTC is a game-based intervention with an *adaptable structure* consisting of 4 stations played in-person and a wrap-up activity at a facilitated environment, and 1 take-home activity. Unique to UTC, games do not have to be played in a specific sequence, they do not have to be completed before playing a new game, nor are they constrained to be completed within a specific time frame. For this evaluation, each game will be “played” for at least 90 minutes, split up into sessions of 30 minutes per game. When combining all gameplay sessions, along with the instructions for the take-away activity and program wrap-up activity, organizations will dedicate approximately 8 hours to the program. The facilitator can alter which games are played during each session to keep youth engaged and prevent burnout. This process will continue until each game is played to completion.

UTC consists of 4 interactive games, 1 warp-up activity, and 1 take-home activity that collectively targets the knowledge, skills, and social supports identified in the conceptual model. Specifically, *The Sum of the Parts* focuses on adolescent development through trivia-style activities addressing physical, emotional, and social changes; *Tapped In* teaches youth how to identify and access credible sources of health information; *More Than Words* provides opportunities to recognize and practice effective communication skills through interactive scenarios; and *Stop-Think-Act* engages youth in structured problem-solving and decision-making exercises using realistic situations. The take-home activity, *Making Connections*, encourages youth to initiate conversations with trusted adults and strengthen supportive adult relationships. Across activities, youth are provided opportunities to practice skills, receive feedback, discuss consequences of decisions, and engage in peer learning within a facilitated environment. Table 2 provides a description of each game and activity.

Depending on the number of youth, staff, and space available, a site can have multiple groups playing different games or multiple groups playing the same game (at different tables) simultaneously. The intervention can be facilitated over the course of 2 days or up to 10 weeks depending on the setting and the participants’ needs.

**Table 2:** UTC Game alignment with objectives, behavior change techniques, and conceptual model constructs.

Game or activity	Objective and description	Behavior change techniques (conceptual model constructs)
Making Connections (activity)	To make safe connections with trusted adults. Overview of instructions: 1. Each youth gets a zipper bag with instructions, conversation cards, safety tips, and contact information card. 2. Find a trusted adult to connect with. 3. Pick a question card and ask the adult the question. Then the adult asks you the second question on the card. 4. Write down the adults’ contact info on the contact card to connect in the future if needed. 5. Give the adult the card to talk with other youth in the community.	Social support (EE^a^, SE^b^) Behavioral practice/rehearsal (SE)
The Sum of the Parts (game; similar to a trivia game)	To learn about changes to the mind and body as you grow/develop. Overview of instructions: 1. Roll picture dice. 2. Draw a card from the deck matching the image on the dice; pass the card to a neighbor to read out loud. 3. Answer the card question correctly. Topics of card decks: (1) Anatomy and physiology, (2) Mind and emotions, and (3) Social aspects and interactions	Instruction on how to perform the behavior Social support (AC^c^, SE) Information about health consequences (EE) Behavior substitution Behavioral practice/rehearsal (SE) Pros and cons (EE) Imaginary reward/punishment (EE, SE) Vicarious consequences (EE, SE)
Tapped In (game; similar to a board game)	To identify and practice accessing credible sources of information. Overview of instructions: 1. Roll numbered dice and move playing piece. 2. Draw a card from the deck matching the colored space the piece landed on; pass the card to a neighbor to read out loud. 3. Answer the card question correctly. If piece landed on a black/challenge space, complete the Challenge on the card to practice accessing sources of information. Topics of card decks: (1) Healthcare providers, (2) Schools, (3) Technology, and (4) Community	Instruction on how to perform the behavior Social support (AC, SE) Problem solving (SE) Behavioral practice/rehearsal (SE) Credible source (EE, AC) Imaginary reward/punishment (EE) Vicarious consequences (EE)
More Than Words (game; similar to a board game with activity cards)	To identify and practice using effective communication skills. Overview of instructions: 1. Roll colored dice and move playing piece. 2. Draw the cards from the space they landed on and identify what communication skills or styles were used, if any. (Each space contains cards with parts of a script. The script will develop with each move. The final script will vary by paths taken and communication skills and styles used or not used.) Repeat steps 1-2 until reaching a final script. 3. Draw and answer debrief questions. 4. Players rewrite the script in their own words.	Instruction on how to perform the behavior Social support (SE) Problem solving (SE) Information about health consequences (EE) Demonstration of the behavior Behavioral practice/rehearsal (SE) Generalization of target behavior Vicarious consequences (EE)
Stop-think-act (game; similar to a life-size board game)	To think critically about scenarios and problem solve to make healthy decisions. Overview of instructions: 1. Youth work together as a group to complete. Each youth is assigned a place on the board to begin (and instructions for moving to new sections) based on the number of players. 2. Player in section 1: Draw and read a scenario card out loud. 3. Players in section 2: Identify different decisions for the scenario. 4. Players in section 3: Write down the pros and cons for each decision. 5. Players in section 4: Identify a local youth-friendly provider that youth could talk to if they were in that situation. 6. Player in section 5: Review the pieces and decide what the healthiest decision is. 7. All participants: Answer discussion questions.	Instruction on how to perform the behavior Social support (AC, SE) Problem solving Behavioral practice/rehearsal (SE) Pros and cons (EE) Imaginary reward/punishment (EE) Vicarious consequences (EE)
Program Wrap Up (activity; similar to completing a puzzle)	To recap and reflect on the knowledge and skills learned through participating in UTC. Overview of instructions: 1. Upon completing all 4 games, the facilitator will gather all of the youth, pass out all game/logo stickers and the wrap up card. 2. Sum of the Parts: you learned information and practiced skills, so that you understand more about your body and your emotion. Tell me something you learned in this game? (Answer). Place the Sum of the Parts sticker in its place on your card. 3. Tapped In: you learned information and practiced skills, so that you understand how to access information about your health. Tell me something you learned in this game? (Answer). Place the Tapped In sticker in its place on your card. 4. More Than Words: you learned information and practiced skills, so that you understand more about how to communicate with your peers, partners, and/or trusted adults. Tell me something you learned in this game? (Answer). Place the More Than Words sticker in its place on your card. 5. Stop, Think, Act: you learned information and practiced skills, so that you understand how to solve problems and make healthy decisions. Tell me something you learned in this game? (Answer). Place the Stop, Think, Act sticker in its place on your card. Goal setting: turn the program wrap up card over a write 2 strategies/goals they want and will commit to in the next 2 weeks.	EE, AC, SE

^a^EE: expectations and expectancies.

^b^SE: self-efficacy.

^c^AC: perceived trusted adult connections.

### Fidelity

UTC fidelity will be enhanced through standardized facilitator training and ongoing technical assistance by the implementation team. Fidelity will be tracked using facilitator logs developed by the implementation team. The facilitator log consisted of session date, minutes/hours the session lasted, location of the session, which games were played during the session, notes about the quality of the session (activities went well or did not go well), and notes on youth engagement after each event to capture implementation logistics and “real-time” notes.

### Comparison Group

Youth in the control group received “business-as-usual” for each corresponding control organization, which consisted of a broad range of health-related topics, including physical activity, career education, academic enrichment, mental health awareness, and awareness of community resources. The purpose of this trial is to evaluate UTC as a complete intervention package compared with programming routinely available in community settings. The study was not designed to isolate the independent effects of game-based delivery from intervention content. The “business-as-usual” control did not include the GBL activities or associated skill-building exercises incorporated within UTC.

### Sample Size Calculation

#### Primary Outcomes

Our main primary outcome is intention to delay sexual intercourse for those who completed UTC vs the control group at posttest and at 3-month follow-up. We conducted a power analysis based on this primary outcome. A minimum of 900 participants (in total) achieves 81% power to detect a difference of 0.5 in the change of mean score on sexual intention between baseline and posttest/follow-up, with an SD of 2.5, using a 2-sided *t* test with a significance level of 0.05. In addition, we also estimated the intraclass correlation coefficient at 0.002. Accounting for a 22% attrition rate, at least 1150 participants will be recruited throughout the study to ensure a sufficient minimum sample size of 900. We predicted 22% attrition, as this rate aligns with the average attrition rate for first short term follow-up in previous pregnancy prevention program evaluations [30]. We will use this sample size to analyze additional primary outcomes comparing youth who completed UTC vs those that have not at posttest and 3-month follow-up: (1) communication skills, (2) decision-making skills, (3) skills related to accessing sexual health information and resources, and (4) perception of connection to trusted adults.

#### Secondary Outcomes

Our secondary outcomes, comparing youth who completed UTC vs those that have not at 9-month follow-up, include (1) intention to delay sexual intercourse, (2) communication skills, (3) decision-making skills, (4) skills to access sexual health information and resources, (5) perception of connection to trusted adults, and (6) intention to use condoms or contraceptives when sexually active. As most of the secondary outcomes are also primary outcomes, but at different time points, we will analyze these outcomes using the sample size calculated for the main primary outcome.

### Data Collection Methods

#### Study Procedures

Youth will use their personal or site-provided electronic devices—such as a cell phone, tablet, or computer—to scan a QR code or input a link to access the REDCap system [38,39] and complete their eligibility screening, assent form, and contact information form. While still in the REDCap system and prior to initiating the program, the participants will complete a baseline survey on an electronic device. After completing the treatment or control program, youth will complete an immediate post-survey using the same electronic device. Participants will receive a REDCap survey via text, email, or mail to complete their electronic 3-month and 9-month follow-up surveys. Participants will be compensated US $10, US $15, US $25, and US $50 for each subsequent survey.

#### Survey Instrument

#### Overview

We compiled a developmentally appropriate survey using established scales to measure demographic characteristics, intention to delay sex, communication skills, decision-making skills, skills to access sexual health information and resources, perception of connection to trusted adults, and intention to use condoms and contraceptives when sexually active [1,27,40-59]. The survey was reviewed by adolescent pregnancy content experts and community stakeholders. Specifically, 3 content experts reviewed the instrument for construct relevance, content accuracy, technical flaws, grammar, appropriateness, and readability [60]. Cognitive interviews were conducted with Texas youth aged 11-14 years (grades 6th-8th) to evaluate the survey instrument’s readability and comprehension. See Multimedia Appendix 1 for the full survey instrument.

#### Demographic Characteristics

Participant characteristics will be measured using self-reported items related to age, grade level, race/ethnicity, sex, primary language used at home, housing situation, access to the internet at home, education level of mother and father, frequency of attendance at religious services, educational goals, and average of school grades. In addition, we will measure whether the participant’s mother was a teen parent.

#### Intentions

Items measuring intention to delay sexual activity were adapted from previously published measures and refined using exploratory factor analysis (EFA) with pilot data to examine the underlying factor structure [46]. We will use 5 items to measure intention to delay sexual intercourse at different time points. For example, “Starting today, will you wait to have sex until you are in a long-term relationship? Some people call this ‘making love’ or ‘doing it.’” The wording of this item and the definition for “sex” was refined based on the pilot testing cognitive interviews with middle school–aged youth to ensure readability, comprehension, and developmentally appropriate terminology. Higher scores will indicate a stronger intention to delay sexual intercourse. In addition to having a continuous variable for intention to delay sexual intercourse, we will create a dichotomous variable for a sensitivity analysis by calculating individual participants’ mean from the 5 items and recoding the means to the following outcomes, 0=does not intend to have sex (Mean=1, 2, 3) and 1=intend to have sex (mean=4, 5).

We will also use 3 items to measure intention to use condoms and contraceptives (eg, “do you plan to use [or have your partner use] a condom when you have sex?”). Items measuring intention will use a 5-point Likert scales (0=no, definitely not, 4= yes, definitely). Higher scores will indicate a stronger intention to use condoms and contraceptives when sexually active. Items measuring intentions to use protection were adapted from existing instruments [44,46-48].

#### Communication Skills

Items measuring communication skills were adapted from existing measures and modified to align with the intervention’s theory of change [27,50,52]. Self-efficacy related to communication skills will consist of 2 subscales: communication skills regarding sexual behaviors, and skills regarding conflict resolution. Responses for these subscales will be measured using a 5-point Likert scale (1=strongly disagree; 4=strongly agree). Communication skills related to sexual behaviors will include 4 items with higher values indicating higher self-efficacy related to communicating about sexual behaviors. The subscale measuring skills related to conflict resolution will include 4 items with higher values indicating higher-self efficacy related to skills in resolving relationship conflict.

#### Decision-Making Skills

Items measuring decision-making skills related to pregnancy prevention and decreased risk for STIs were adapted from prior instruments and refined using EFA results with pilot data [12,29]. Decision-making skills will be divided into 2 subscales: self-efficacy in decision-making regarding sexual behaviors, and expectancies and expectations regarding sexual health outcomes. The self-efficacy subscale will include up to 5 items with higher values indicating higher self-efficacy related to skills in making decisions about behaviors leading to pregnancy prevention and decreased risk for STIs. The expectancies and expectations subscale will include up to 7 items with higher values indicating positive expectancies and expectations related to making decisions for behaviors leading to pregnancy prevention and decreased risk for STIs. Responses for these subscales will be measured using a 5-point Likert scale (1=strongly disagree; 4=strongly agree).

#### Skills in Accessing Sexual Health Information and Resources

Items measuring ability to access information were adapted from multiple sources and refined through EFA with pilot data [44,50,53-56]. This scale will include 11 items with higher values indicating higher self-efficacy related to skills in accessing sexual health information and resources. Responses for these subscales will be measured using a 5-point Likert scale (1=strongly disagree; 4=strongly agree).

#### Perception of Connection to Trusted Adults

Items measuring perceptions of connection to trusted adults were adapted from prior research [27,28]. We will measure participants’ perception of connection to trusted adults by assessing their perception of having an adult to talk to about puberty, dating, pregnancy prevention, and STI prevention. Responses for this scale will be measured using a 5-point Likert scale (1=strongly disagree; 4=strongly agree). This scale will include 12 items with higher scores indicating a stronger perception of connection to trusted adults.

### Outcome Coding

An EFA will be conducted to assess each respective scale and subscale survey items for convergent and construct validity. Variables will be retained in a scale if the pattern/structure coefficient was greater than |0.4|. The scale selected in the EFA will be assessed to ensure the factor analysis has identified a cohesive scale identifying a single underlying construct. Cronbach α will be calculated. Based on the EFA results, a mean of all retained items will be calculated.

### Participant Retention

Multiple retention tools will be sent to participants to increase engagement, including personalized birthday cards with reminders of the next study activity, stickers with the study logo, and magnets with the study contact information. To minimize adolescent participant attrition, we will also implement an evidence-based retention model, the engagement, verification, maintenance, and confirmation (EVMC) protocol [61]. The EVMC protocol provides a stepwise process to reach participants regardless of mobility rates, quickly detect inaccurate contact information, and create multiple avenues to relocate participants [62]. For *engagement*, participants will complete a contact information form immediately after enrolling in the study. The form will collect participants’ cell phone numbers, email addresses, and mailing addresses, as well as ask participants to provide the email address, phone number, and mailing address of a trusted adult. Next, *verification* will be used on an “as needed” basis by contacting trusted adults to verify participant contact information if a participant is not completing survey activities. For the *maintenance* phase, we will send messages each month to participants with cell phones and email addresses reminding them when the next study activity will occur. We will also ask participants to *confirm* their contact information within the same monthly text and email messages used in the maintenance phase.

### Data Security and Storage

REDCap [38,39] will be used to complete and store data related to research activities including eligibility screening, assent forms, contact information, surveys and responses, and text and email reminders sent to participants. REDCap is a secure web-based data management platform designed exclusively to provide an intuitive interface for data capture in 21 Code of Federal Regulations Part 22, Federal Information Security Act, and Health Insurance Portability and Accountability Act–compliant environments. By using REDCap, we assure procedures for obtaining consent, data collection, and storage address confidentiality and privacy matters for study data and information collected. The REDCap system is housed in the CCHMC Information Services for Research (IS4R) on premises server system, which provides both physical and network security. Access to this system is limited to authorized staff only, with credentials maintained by CCHMC. Individual computer systems used by the evaluation team comply with CCHMC standards for device encryption and security. Within the REDCap system, each participant will be assigned a unique study ID and contact information will be stored in a REDCap form separate from their survey responses. Links between individual-level data and participant study IDs will be destroyed once the study is completed. The deidentified data will be stored on the IS4R server system. Per the data sharing agreement between the implementation and evaluation teams, CCHMC will provide TAMU appropriate scientific data and any additional metadata. A releasable quantitative database will be produced and completely deidentified in accordance with the data-sharing agreement prior to data sharing with TAMU. The database will not contain any site or participant identifiers. The evaluation team will develop a data dictionary consisting of all data elements and their definitions.

### Statistical Methods

#### Overview

Descriptive analysis (frequencies, means, and SD) will be performed to describe participant characteristics, demographics, primary outcomes, and secondary outcomes. The reported rate of intention to delay sex (primary outcome) after treatment will be compared to the control group using a chi-square test or Fisher exact test, depending on the distribution of the data. A 2-sample *t* test will be used to detect the difference in mean scores between the treatment and control groups.

Linear regression models will be performed for continuous outcomes, and logistic regression models will be used for categorical outcomes. Random-effects models (mixed models) will be used to consider the cluster effect (ie, the correlation between measurements from participants within the same cluster). Under a low-attrition randomized controlled trial (RCT), we plan to assess between-group differences at the corresponding outcome time point for each contrast. If the final attrition exceeds the What Works Clearinghouse’s threshold for low attrition, we will assess and establish baseline equivalence between treatment and control groups for each analytic sample at each outcome time point.

To account for possible covariate imbalance that could occur from our block randomization, equivalence will be assessed on key demographic and preintervention covariates using standardized mean differences. If equivalence falls between 0.05 and 0.25 SD, we will include the covariates in the impact models to statistically adjust for residual imbalance. If standardized mean difference is >0.25 SD and cannot be adjusted, the analysis may not meet standards for causal inference. All tests will be 2-sided with a statistical significance level of 0.05 (ie, *P*<.05 will be considered statistically significant). All analyses, including the regression models, will be performed using SAS (version 9.4; SAS Institute Inc).

#### Covariates

We will include the following variables as covariates: age or grade level, race/ethnicity, sex, primary language used at home, housing situation, access to internet at home, education level of mother and father, average of school grades, and whether the participant’s mother was a teen parent. Variable selection will be performed, when necessary, under stepwise criteria. A value of *P*<.05 and *P*>.15 will be used for inclusion and exclusion thresholds.

#### Missing Data

For missing outcome data, we will use case deletion, removing observations with incomplete outcome variables from the analysis. We will assume that missingness is random and our primary analyses remain unbiased under the assumption. For missing covariates, multiple imputation will be performed using predictive modeling, including an indicator variable for intervention status and all the covariates that are used for statistical adjustment in the impact estimation model to replace missing values based on available data. The imputation model includes the outcome when imputing missing baseline data. Sensitivity analyses will be conducted to assess the impact of missing data assumptions on our results. By integrating case deletion for missing outcomes and imputation for missing covariates, we aim to minimize bias while maximizing the use of available data.

#### Sensitivity Analysis

A sensitivity analysis will be conducted to assess the impact of assessing intention to delay sexual intercourse as a continuous variable (linear regression) vs a dichotomous variable (logistic regression). Our primary contrast will be analyzed with intention to delay sexual intercourse as a continuous variable, with the dichotomous variable serving as the sensitivity analysis. A sensitivity analysis will also be conducted to assess the impact of missing data assumptions on our results.

### Data Safety and Monitoring

Because the behavioral intervention and study activities meet the requirements for minimal risk, we will monitor adverse events and will report events to the evaluation team’s institutional review board consistent with Cincinnati Children’s Research Foundation policy. The principal investigator (BR) will be responsible for participant safety and monitoring. All site facilitators will be trained in reporting all adverse events to the principal investigators or designated research coordinators. Participants will also be encouraged to contact the evaluation team with any questions or concerns and will receive the team’s contact information (study email and phone number) at enrollment. No interim analyses or formal stopping guidelines were planned due to the minimal risk nature of the behavioral intervention.

### Ethical Considerations

Ethics approval for this study was obtained from the CCHMC Institutional Review Board (IRB #2022-0471). A waiver of parental permission was requested and approved for this study. This is a minimal risk study and the waiver of parental consent will reduce bias in the enrollment of the study participants and has been granted for similar studies. Parents will be informed of the research through the study information sheet; however, due to the design of the program there is not a natural way for parents to provide parental permission nor to complete the informed consent process. All participants provided informed assent before enrollment. All study activities were optional, and participants were informed of their ability to opt out at any time. Access to participant data was restricted to authorized study personnel only and maintained with REDCap, secure web-based data management platform. A deidentified dataset was created prior to analysis to protect confidentiality and privacy of all participants. Participants received study compensation in the form of digital payments or gift cards for each completed study survey, not exceeding US $100 total for full study completion. These payment structures are set in accordance with CCHMC compensation guidelines and policies for human subjects research with minors.

## Results

Funding was secured in September 2021. Recruitment began in June 2023. The first participant was enrolled in the study on June 15, 2023, and the last participant was enrolled on May 31, 2025. Follow-up data collection was completed on April 1, 2026. As of July 2026, all study activities have concluded. While 46 sites were planned for recruitment, 53 sites were randomized to ensure we met our participant recruitment goal. Of the 53 sites, 13 sites withdrew, and a total of 40 sites enrolled participants into the study. For this study, 1338 participants were enrolled and data from 1231 participants were analyzed. A final report will be submitted to PREIS by September 2026. Results will be presented at scientific conferences and published in peer-reviewed journals.

The study was initially planned for implementation in Texas. To support recruitment and implementation feasibility, additional sites were enrolled in Alabama and Illinois. While 46 sites were originally planned, 53 sites were randomized to meet recruitment goals, with 40 sites ultimately enrolling participants.

## Discussion

This study will provide the first rigorous evaluation of UTC, a novel game-based intervention for adolescent pregnancy prevention. Consistent with the intervention’s conceptual model, we hypothesize that participants receiving UTC will demonstrate an increase in intention to delay sexual intercourse, communication skills, decision-making skills, ability to access sexual health information and resources, and perceptions of connection to trusted adults compared with youth receiving business-as-usual programming. If effective, UTC will provide evidence supporting the use of GBL approaches in community settings to strengthen intermediate outcomes related to adolescent pregnancy prevention among underserved youth. Moreover, data from this study will allow us to refine and optimize our conceptual model regarding UTC’s impact on these outcomes and how they may serve as paths to influence behavior. Overall, this evaluation data will contribute to strengthening the evidence-base for adolescent pregnancy prevention.

Prior federally funded adolescent pregnancy prevention evaluations have demonstrated modest effects on behavioral outcomes while highlighting the importance of examining intermediate outcomes that often lead to behavior change [25,26,31,63]. Building on these findings, the adolescent pregnancy prevention field has called on researchers to develop and test innovative approaches, examine intermediate protective factors, and begin to evaluate the mechanisms (the *how* and *why*) through which interventions produce their effects [10,64]. Our study responds to these calls by evaluating a novel, GBL intervention and explicitly measuring its impact on theory-driven intermediate outcomes, thus beginning to observe the possible mechanisms of action. This study will provide evidence regarding the potential of a GBL intervention to influence theory-driven, protective factors among middle school–aged youth, establishing an innovative approach to adolescent pregnancy prevention before sexual behaviors occur.

Aligned with the field’s calls to test innovative delivery approaches, UTC incorporates innovative implementation features. Unlike health curricula that require content to be delivered in a restricted sequence, UTC was intentionally designed to allow facilitators to adapt the order and pacing of the games to fit the needs and interests of youth. Intervention fidelity is maintained through participants playing each core game and activity rather than adherence to a specific content sequence. The facilitators’ ability to tailor the content order may enhance the feasibility, acceptability, adoption, and scalability of UTC across community organizations while simultaneously preserving UTC’s core functions. Rather than a threat to fidelity, implementation variation is a deliberate, intended design feature that is expected to increase the potential for successful real-world implementation.

In addition to the flexibility of the intervention implementation, there are multiple evaluation design strengths. First, our multisite, clustered RCT design reduces selection bias and allows the intervention to be tested across a multitude of community settings, enhancing the rigor and the study’s external validity. We also minimized the possibility of intervention contamination by randomizing at the site level. In alignment with the PREIS Notice of Funding Opportunity requirements, the evaluation is being conducted by an independent third-party evaluation team. To ensure inclusion in the US Department of Health and Human Services TPP Evidence Review, the evaluation also includes prespecified outcomes and analytic procedures, including multiple sensitivity analyses [65]. These practices reduce the risk of analytic bias and strengthen the finding’s transparency and credibility. Furthermore, we adhered to established best practices for rigorous evaluation research by registering the protocol on ClinicalTrials.gov prior to participant recruitment and enrollment, thereby promoting transparency and accountability and reducing the potential for selective outcome reporting [66].

This study will have several limitations that should be considered. First, outcomes are based on self-reported data and may be subject to recall and social desirability bias. Second, because of implementation logistics, it is not possible to blind participants, facilitators, the implementation team, or evaluation personnel to study assignment. However, the study statistician will remain blinded during primary analyses to reduce bias in analytic decisions, interpretation, and reporting [67]. Third, the study focuses on intermediate outcomes rather than behavioral outcomes because the middle school population is expected to have limited variance in sexual behavior. This lack of variance reduces the ability to detect meaningful intervention effects on behavioral outcomes. Finally, the 9-month follow-up period will likely result in participant attrition, potentially affecting study power and introducing bias if loss to follow-up differs across groups.

Future research should assess whether changes in intermediate outcomes lead to behavioral outcomes, such as delayed sexual initiation, contraceptive use, and condom use. Additional evaluations are required to assess the effectiveness of UTC in other settings (eg, schools) and priority populations (eg, rural populations and high school youth). UTC evaluations should also examine the mechanisms (eg, skills in decision-making, communication, and accessing information and resources) through which it influences behavior. Results will be disseminated through (1) an evaluation report to the funder, (2) presentations at international and national scientific conferences, and (3) peer-reviewed publications. In addition, findings will be shared with participating organizations, implementation partners, and community stakeholders via presentations and summary reports. Dissemination efforts will focus on translating study findings into practical recommendations for adolescent pregnancy prevention practitioners and informing future adaptation, implementation, and evaluation of UTC and other GBL interventions. Lessons learned regarding intervention delivery, participant engagement, and evaluation methodology will also be shared to support researchers and organizations developing and evaluating innovative adolescent pregnancy prevention programs.

## Appendix

Multimedia Appendix 1 Survey instrument.

## Abbreviations

BCT: behavior change technique
CBO: community-based organization
CCHMC: Cincinnati Children’s Hospital Medical Center
CCTST: Center for Clinical and Translational Science and Training
CIRTification: Community Involvement in Research Training
cRCT: clustered randomized controlled trial
EFA: exploratory factor analysis
EVMC: engagement, verification, maintenance and confirmation
GBL: game-based learning
HCD: human-centered design
IS4R: CCHMC Information Services for Research
iTP3: Innovative Teen Pregnancy Prevention Programs
PREIS: Personal Responsibility Education Program Innovative Strategies
PREP: Personal Responsibility Education Program
RCT: randomized controlled trial
SCT: social cognitive theory
STI: Sexually transmitted infection
TAMU: Texas A&M University
TPP: Teen Pregnancy Prevention
UTC: Using the Connect

## Data Availability

The datasets generated and analyzed during the study will not be publicly available because participants were not consented for public data sharing and the study includes minors and sensitive adolescent sexual health information. Requests for deidentified data may be considered by the study investigators on a case-by-case basis, subject to institutional review, applicable regulations, data use agreements, and participant privacy protections.
