# Supplementary material for: Game-Based Intervention for Adolescent Pregnancy Prevention: Protocol for a Multisite Randomized Controlled Trial Evaluation
Source: JMIR Res Protoc. 2026 Oct 8;15:e88047. doi: 10.2196/88047 (PMC13691462; doi:10.2196/88047)
Supplement: Supplementary file 1 [file resprot_v15i1e88047_app1.docx]

**SURVEY INSTRUMENT**

| **CONSTRUCT & MEASURES** | | **TIMEPOINTS** | | | |
| --- | --- | --- | --- | --- | --- |
| **Survey Question** | **Response Options** | **Pre-Survey** | **Post-Survey** | **3-Month Survey** | **9-Month Survey** |
| ***PREIS Middle School Entry Questions [1-2]*** | | | | | |
| How old are you? | 10-16 | ✓ | ✓ | ✓ | ✓ |
| What grade are you in? (If you are currently on vacation or in summer school, indicate the grade you will be in when you go back to school.) | 5^th^-9^th^  My school does not assign grade levels  I am not currently enrolled in school | ✓ | ✓ |  |  |
| When you are at home or with your family, what language or languages do you usually speak? | English/Spanish/Other | ✓ | ✓ |  |  |
| Are you Hispanic or Latino? | Yes/No | ✓ | ✓ |  |  |
| What is your race? | American Indian or Alaska Native  Asian  Black or African American  Native Hawaiian or other Pacific Islander  White or Caucasian  Other (specify) | ✓ | ✓ |  |  |
| What is your sex? | Male/ Female | ✓ | ✓ | ✓ | ✓ |
| Are you currently…? | Living with family / In foster care, living with a family / In foster care, living in a group home / Couch surfing or moving from home to home / Living outside, in a tent city or homeless camp, in a car, in an abandoned vehicle or in an abandoned building / Staying in an emergency shelter or transitional living program / Staying in a hotel or motel / In juvenile detention center, juvenile group home, and/or under the supervision of a probation officer / None of the above | ✓ | ✓ |  |  |
| ***Youth demographics [2-4]*** | | | | | |
| Do you have internet at home? | Yes/No | ✓ | ✓ | ✓ | ✓ |
| [if no to question above] Where do you usually get internet? | Smartphone or tablet with internet  School  Friend’s House  Family member’s House  Library  Community Center, Youth Center  Other (specify) | ✓ | ✓ | ✓ | ✓ |
| What level of education did your mother complete? | Completed some high school  Graduated High School (or completed GED)  Graduated College  Unknown | ✓ | ✓ | ✓ | ✓ |
| What level of education did your father complete? | Completed some high school  Graduated High School (or completed GED)  Graduated College  Unknown | ✓ | ✓ | ✓ | ✓ |
| How many times have you been to a religious service (like church) in the past 12 months? | Once a week or more / two times a month / once a month / A few times / Never | ✓ | ✓ | ✓ | ✓ |
| What are your education goals? | I plan to…  Complete at least a 4-year college degree (Like a Bachelor’s degree) / Complete a 2-year college degree (Like an Associate’s degree) / Complete career, vocational or trade training (Like a dental hygienist, mechanic, paramedic) / Graduate high school/ I do not plan to finish high school | ✓ | ✓ | ✓ | ✓ |
| What are your school grades? | Mostly A’s/ Mostly B’s/Mostly C’s/ Mostly D’s /  Mostly F’s | ✓ | ✓ | ✓ | ✓ |
| Was your mother a teen parent?  Select ‘yes’ if you know your mother had a baby when she was 19 years old or younger | Yes / No / I don’t know | ✓ | ✓ | ✓ | ✓ |
| ***Intention to delay sex [4-8]*** | | | | | |
| If you have the chance, do you plan to have sex in the next year?  Some people call this ‘making love’ or ‘doing it’. | 5 point scale: no, definitely not; no, probably not; maybe; yes, probably; yes, definitely | ✓ | ✓ | ✓ | ✓ |
| Starting today, will you wait to have sex until you finish high school? | 5 point scale: no, definitely not – yes, definitely | ✓ | ✓ | ✓ | ✓ |
| Starting today, will you wait to have sex until you are in a long-term relationship? | 5 point scale: no, definitely not – yes, definitely | ✓ | ✓ | ✓ | ✓ |
| ***Intentions to use condoms / contraceptives [5-10]*** | | | | | |
| Starting today, do you plan to use (or have your partner use) a condom when you have sex? | 5 point scale: no, definitely not – yes, definitely | ✓ | ✓ | ✓ | ✓ |
| Starting today, do you plan to use (or have your partner use) birth control (like the pill, IUD, or implant) when you have sex. | 5 point scale: no, definitely not – yes, definitely | ✓ | ✓ | ✓ | ✓ |
| ***Alternative behaviors [11-14]*** | | | | | |
| Have you ever had a boy/ girlfriend? | Yes/No | ✓ | ✓ | ✓ | ✓ |
| Do you currently have a boy/ girlfriend? | Yes/No | ✓ | ✓ | ✓ | ✓ |
| Have you and a boy/ girlfriend ever held hands? | Yes/No | ✓ | ✓ | ✓ | ✓ |
| Have you and a boy/ girlfriend ever kissed? | Yes/No | ✓ | ✓ | ✓ | ✓ |
| Have you and a boy/ girlfriend ever cuddled? | Yes/No | ✓ | ✓ | ✓ | ✓ |
| Have you and a boy/ girlfriend told each other that you love each other? | Yes/No | ✓ | ✓ | ✓ | ✓ |
| Have you ever spent time alone with a boy/ girlfriend? | Yes/No | ✓ | ✓ | ✓ | ✓ |
| *If participant is 14 years of age or older*  Have you ever touched someone’s private parts with their clothes on? | Yes/No | ✓ | ✓ | ✓ | ✓ |
| *If participant is 14 years of age or older*  Have you ever touched someone’s private parts without their clothes on? | Yes/No | ✓ | ✓ | ✓ | ✓ |
| ***Behavioral Capability [9-10, 15-16]*** | | | | | |
| Do you think…if condoms are used correctly and consistently, they can lower the risk of getting a sexually transmitted infection (STI/STD)? | Yes/No | ✓ | ✓ | ✓ | ✓ |
| Do you think… If birth control pills are used correctly and consistently, they can lower the risk of getting a sexually transmitted infection (STI/STD) | Yes/No | ✓ | ✓ | ✓ | ✓ |
| Which of these are ways to lower the risk of getting a sexually transmitted infection (STI/STD)?  *Select all that apply* | Waiting until you are in a long term relationship to have sex /Using condoms  Taking antibiotics/Drinking lots of water/Showering after sex/Avoiding kissing / Shaving genital area | ✓ | ✓ | ✓ | ✓ |
| Which of these are ways a person can get pregnant, or get someone else pregnant?  *Select all that apply* | Having sex /Touching private parts with hands/Touching private parts over clothes /Kissing /Cuddling with clothes on/ Cuddling without clothes on | ✓ | ✓ | ✓ | ✓ |
| Which of these are ways a person can prevent pregnancy?  *Select all that apply* | Not having sex / Wearing a condom during sex/ Using birth control (like the pill, IUD, or implant) /  Peeing after sex/ Showering after sex | ✓ | ✓ | ✓ | ✓ |
| Which of these are places you can get condoms?  *Select all that apply* | Grocery store/ Gas station or Convenience store / Doctor or nurse / A trusted adult / Pharmacy | ✓ | ✓ | ✓ | ✓ |
| Which of these are people who can help you or your partner get birth control (like the pill, IUD or implant)?  *Select all that apply* | School nurse/ A trusted adult / Doctor or nurse/ Pharmacist | ✓ | ✓ | ✓ | ✓ |
| ***Communication skills [10, 12]*** | | | | | |
| Whether or not you have dated, do you agree with the following statement: I can say “no” to a date with someone | 5-point scale: strongly disagree; disagree; unsure; agree; strongly agree | ✓ | ✓ | ✓ | ✓ |
| Whether or not you have dated, do you agree with the following statement: I can break-up with someone | 5-point scale: strongly disagree-strongly agree | ✓ | ✓ | ✓ | ✓ |
| Whether or not you have dated, do you agree with the following statement: I can tell someone I’m dating how I want to be treated | 5-point scale: strongly disagree-strongly agree | ✓ | ✓ | ✓ | ✓ |
| Whether or not you have dated, do you agree with the following statement: I can solve a disagreement with someone I’m dating | 5-point scale: strongly disagree-strongly agree | ✓ | ✓ | ✓ | ✓ |
| Sexual consent means that each person wants to have sex. Do you agree with the following statement: I can give consent out loud to someone I want to have sex with | 5-point scale: strongly disagree-strongly agree | ✓ | ✓ | ✓ | ✓ |
| Sexual consent means that each person wants to have sex. Do you agree with the following statement: I can ask for consent from someone I want to have sex with | 5-point scale: strongly disagree-strongly agree | ✓ | ✓ | ✓ | ✓ |
| I can talk about using condoms with someone I want to have sex with | 5-point scale: strongly disagree-strongly agree | ✓ | ✓ | ✓ | ✓ |
| I can talk about birth control (like the pill, IUD, or implant) with someone I want to have sex with | 5-point scale: strongly disagree-strongly agree | ✓ | ✓ | ✓ | ✓ |
| ***Decision making skills [17]*** | | | | | |
| I can wait to have sex until I am in a long-term relationship | 5-point scale: strongly disagree-strongly agree | ✓ | ✓ | ✓ | ✓ |
| I can wait to have sex until I am able to get condoms | 5-point scale: strongly disagree-strongly agree | ✓ | ✓ | ✓ | ✓ |
| I can wait to have sex until me or my partner are able to get birth control (like the pill, IUD, or implant) | 5-point scale: strongly disagree-strongly agree | ✓ | ✓ | ✓ | ✓ |
| I can wait to have sex until I’m finished with high school | 5-point scale: strongly disagree-strongly agree | ✓ | ✓ | ✓ | ✓ |
| No one can pressure me into having sex | 5-point scale: strongly disagree-strongly agree | ✓ | ✓ | ✓ | ✓ |
| I will not have sex until I find the right person | 5-point scale: strongly disagree-strongly agree | ✓ | ✓ | ✓ | ✓ |
| If my partner or I have sex without birth control (like the pill, IUD, or implant), we might get pregnant | 5-point scale: strongly disagree-strongly agree | ✓ | ✓ | ✓ | ✓ |
| If my partner and I have sex without a condom, we might get a sexually transmitted infection (STI/STD) | 5-point scale: strongly disagree-strongly agree | ✓ | ✓ | ✓ | ✓ |
| If my partner and I have sex without a condom, we might get pregnant | 5-point scale: strongly disagree-strongly agree | ✓ | ✓ | ✓ | ✓ |
| At my age, not having sex is important to be safe and healthy | 5-point scale: strongly disagree-strongly agree | ✓ | ✓ | ✓ | ✓ |
| I want to avoid getting a a sexually transmitted infection (STI/STD) | 5-point scale: strongly disagree-strongly agree | ✓ | ✓ | ✓ | ✓ |
| I do not want to get pregnant or get someone else pregnant right now | 5-point scale: strongly disagree-strongly agree | ✓ | ✓ | ✓ | ✓ |
| ***Accessing information/resources skills [4, 9-10, 18-21]*** | | | | | |
| I can find **helpful** information on the internet | 5-point scale: strongly disagree-strongly agree | ✓ | ✓ | ✓ | ✓ |
| I can find **factual** **health** information on the internet | 5-point scale: strongly disagree-strongly agree | ✓ | ✓ | ✓ | ✓ |
| I can go to the store and buy condoms if I want to | 5-point scale: strongly disagree-strongly agree | ✓ | ✓ | ✓ | ✓ |
| It is embarrassing to buy condoms. | 5-point scale: strongly disagree-strongly agree | ✓ | ✓ | ✓ | ✓ |
| If I decide to have sex, I can have a condom with me | 5-point scale: strongly disagree-strongly agree | ✓ | ✓ | ✓ | ✓ |
| I know where to get birth control (like the pill, IUD, or implant) for me or my partner | 5-point scale: strongly disagree-strongly agree | ✓ | ✓ | ✓ | ✓ |
| When I am ready to have sex, I plan to talk to a nurse or doctor about preventing pregnancy. | 5-point scale: strongly disagree-strongly agree | ✓ | ✓ | ✓ | ✓ |
| I can name two or more places to get help if I feel unsafe with my partner. | 5-point scale: strongly disagree-strongly agree | ✓ | ✓ | ✓ | ✓ |
| I know where to get information to avoid a pregnancy | 5-point scale: strongly disagree-strongly agree | ✓ | ✓ | ✓ | ✓ |
| I have the information I need to avoid a pregnancy | 5-point scale: strongly disagree-strongly agree | ✓ | ✓ | ✓ | ✓ |
| I know where to get information to avoid getting a sexually transmitted infection (STI/STD) | 5-point scale: strongly disagree-strongly agree | ✓ | ✓ | ✓ | ✓ |
| I have the information I need to avoid getting a sexually transmitted infection (STI/STD) | 5-point scale: strongly disagree-strongly agree | ✓ | ✓ | ✓ | ✓ |
| Select the source that has given you the most information about sex in the past 12 months? | Your friends/ Your boy/girlfriend/ Your mother or father/ Siblings or other relatives/ A doctor or nurse/ A teacher / A counselor/ A guest speaker at school / A minister, priest or rabbi/Social Media / Websites/ Books / TV,radio, podcasts / I have not received information about sex in the last 12 months | ✓ | ✓ | ✓ | ✓ |
| ***Perceptions of connection to trusted adult [15, 17, 22]*** | | | | | |
| I have an adult in my life that I can count on | 5-point scale: strongly disagree-strongly agree | ✓ | ✓ | ✓ | ✓ |
| I have an adult in my life who supports me | 5-point scale: strongly disagree-strongly agree | ✓ | ✓ | ✓ | ✓ |
| I have an adult in my life that I can talk to about any issue without judgement | 5-point scale: strongly disagree-strongly agree | ✓ | ✓ | ✓ | ✓ |
| I have talked to a trusted adult (like parents, doctors, teachers) about body changes. | 5-point scale: strongly disagree-strongly agree | ✓ | ✓ | ✓ | ✓ |
| I have talked to a trusted adult (like parents, doctors, teachers) about menstruation / periods. | 5-point scale: strongly disagree-strongly agree | ✓ | ✓ | ✓ | ✓ |
| I have talked to a trusted adult (like parents, doctors, teachers) about going on a date. | 5-point scale: strongly disagree-strongly agree | ✓ | ✓ | ✓ | ✓ |
| I have talked to a trusted adult (like parents, doctors, teachers) about pregnancy. | 5-point scale: strongly disagree-strongly agree | ✓ | ✓ | ✓ | ✓ |
| I have talked to a trusted adult (like parents, doctors, teachers) about sexually transmitted infections (STI/STD). | 5-point scale: strongly disagree-strongly agree | ✓ | ✓ | ✓ | ✓ |
| I have talked to a trusted adult (like parents, doctors, teachers) about using condoms | 5-point scale: strongly disagree-strongly agree | ✓ | ✓ | ✓ | ✓ |
| I have talked to a trusted adult (like parents, doctors, teachers) about me or a partner using birth control (like the pill, IUD or implant). | 5-point scale: strongly disagree-strongly agree | ✓ | ✓ | ✓ | ✓ |
| I have talked to a trusted adult (like parents, doctors, teachers) about consent. | 5-point scale: strongly disagree-strongly agree | ✓ | ✓ | ✓ | ✓ |
| I have talked to a trusted adult (like parents, doctors, teachers) about dating violence. | 5-point scale: strongly disagree-strongly agree | ✓ | ✓ | ✓ | ✓ |
| ***PREIS Middle School Exit Questions [23]*** | | | | | |
| Even if your program didn’t cover this topic would you say that being in the program made you more likely, about the same, or less likely to… Resist or say no to peer pressure | 5-point scale: much more likely – much less likely |  | ✓ |  |  |
| Even if your program didn’t cover this topic would you say that being in the program made you more likely, about the same, or less likely to… Manage your emotions in healthy ways (for example, ways that are not hurtful to you or others)? | 5-point scale: much more likely – much less likely |  | ✓ |  |  |
| Even if your program didn’t cover this topic would you say that being in the program made you more likely, about the same, or less likely to… Make decisions to not use drugs or alcohol? | 5-point scale: much more likely – much less likely |  | ✓ |  |  |
| Even if your program didn’t cover this topic would you say that being in the program made you more likely, about the same, or less likely to… Think about the consequences before making a decision? | 5-point scale: much more likely – much less likely |  | ✓ |  |  |
| Even if your program didn’t cover this topic would you say that being in the program made you more likely, about the same, or less likely to… Make plans to reach your goals? | 5-point scale: much more likely – much less likely |  | ✓ |  |  |
| Even if your program didn’t cover this topic would you say that being in the program made you more likely, about the same, or less likely to… Care about doing well in school? | 5-point scale: much more likely – much less likely |  | ✓ |  |  |
| Even if your program didn’t cover this topic would you say that being in the program made you more likely, about the same, or less likely to… Graduate high school or get your GED? | 5-point scale: much more likely – much less likely |  | ✓ |  |  |
| Even if your program didn’t cover this topic would you say that being in the program made you more likely, about the same, or less likely to… Get more education or training after high school or completing your GED? | 5-point scale: much more likely – much less likely |  | ✓ |  |  |
| Even if your program didn’t cover this topic would you say that being in the program made you more likely, about the same, or less likely to… Get a steady full-time job after school? | 5-point scale: much more likely – much less likely |  | ✓ |  |  |
| Even if your program didn’t cover this topic would you say that being in the program made you more likely, about the same, or less likely to… Save money to get things you want | 5-point scale: much more likely – much less likely |  | ✓ |  |  |
| Even if your program didn’t cover this topic would you say that being in the program made you more likely, about the same, or less likely to… Feel confident about how to open a bank account | 5-point scale: much more likely – much less likely |  | ✓ |  |  |
| Even if your program didn’t cover this topic would you say that being in the program made you more likely, about the same, or less likely to…Feel confident about how to prepare a budget | 5-point scale: much more likely – much less likely |  | ✓ |  |  |
| Even if your program didn’t cover this topic would you say that being in the program made you more likely, about the same, or less likely to… Feel confident about how to track your expenses | 5-point scale: much more likely – much less likely |  | ✓ |  |  |
| Even if your program didn’t cover this topic would you say that being in the program made you more likely, about the same, or less likely to… Understand the costs associated with raising a child | 5-point scale: much more likely – much less likely |  | ✓ |  |  |
| Even if your program didn’t cover this topic would you say that being in the program made you more likely, about the same, or less likely to… Talk with your parents, guardians, or caregivers about things going on in your life? | 5-point scale: much more likely – much less likely |  | ✓ |  |  |
| Even if your program didn’t cover this topic would you say that being in the program made you more likely, about the same, or less likely to… Talk with your parent, guardian, or caregiver about sex? | 5-point scale: much more likely – much less likely |  | ✓ |  |  |
| Even if your program didn’t cover this topic would you say that being in the program made you more likely, about the same, or less likely to… Better understand what makes a relationship healthy? | 5-point scale: much more likely – much less likely |  | ✓ |  |  |
| Even if your program didn’t cover this topic would you say that being in the program made you more likely, about the same, or less likely to…  Resist or say no to someone if they pressure you to participate in acts, such as kissing, touching private parts, or sex? | 5-point scale: much more likely – much less likely |  | ✓ |  |  |
| Even if your program didn’t cover this topic would you say that being in the program made you more likely, about the same, or less likely to… Talk to a trusted person/adult (for example, a family member, teacher, counselor, coach, etc.) if someone makes you uncomfortable, hurts you, or pressures you to do things you don’t want to do? | 5-point scale: much more likely – much less likely |  | ✓ |  |  |
| Even if you didn’t attend all of the sessions or classes in this program, how often in this program… Did you feel interested in program sessions and classes? | 4-point scale: all of the time-none of the time |  | ✓ |  |  |
| Even if you didn’t attend all of the sessions or classes in this program, how often in this program… Did you feel the material presented was clear? | 4-point scale: all of the time-none of the time |  | ✓ |  |  |
| Even if you didn’t attend all of the sessions or classes in this program, how often in this program… Did discussions or activities help you to learn program lessons? | 4 point scale: all of the time-none of the time |  | ✓ |  |  |
| Even if you didn’t attend all of the sessions or classes in this program, how often in this program… Did you have a chance to ask questions about topics or issues that came up in the program? | 4-point scale: all of the time-none of the time |  | ✓ |  |  |
| Even if you didn’t attend all of the sessions or classes in this program, how often in this program… Did you feel respected as a person? | 4-point scale: all of the time-none of the time |  | ✓ |  |  |
| Thinking about the program, how satisfied are you with… The amount of information you received about abstaining from sex (choosing not to have sex)? | 4-point scale: very satisfied-not at all satisfied |  | ✓ |  |  |
| Thinking about the program, how satisfied are you with… The amount of information you received about condoms and birth control? | 4 point scale: very satisfied-not at all satisfied |  | ✓ |  |  |

**SURVEY INSTRUMENT REFERENCES**

[1] Family & Youth Services Bureau. Participant Entry Survey Middle School Mathematica. <https://www.prepeval.com/DataCollection/PREIS-Tribal_MS_Entry_Survey_edited_01182021.pdf>, (accessed 28 July 2026).

[2] Lenhart A, Madden M, Hitlin P. Youth are leading the transition to a fully wired and mobile nation. Washington, DC: Pew Internet & American Life Project; 2005. <https://www.pewresearch.org/internet/2005/07/27/part-2-conditions-of-internet-use/>, (accessed 28 July 2026).

[3] United States Census Bureau. About educational attainment. U.S. Department of Commerce. <https://www.census.gov/topics/education/educational-attainment/about.html>, (accessed 28 July 2026).

[4] Potter SC, Coyle KK, Glassman JR, Kershner S, Prince MS. It's Your Game...Keep It Real in South Carolina: A group randomized trial evaluating the replication of an evidence-based adolescent pregnancy and sexually transmitted infection prevention program. Am J Public Health. 2016;106(S1):S60-S69. doi:10.2105/AJPH.2016.303419.

[5] Coyle K, Anderson P, Laris B, Unti T, Franks H, Glassman J. Evaluation of It's Your Game: Keep It Real in Houston, TX: Final Report. Houston, TX; 2016.

[6] Markham CM, Peskin MF, Shegog R, et al. Behavioral and psychosocial effects of two middle school sexual health education programs at tenth-grade follow-up. J Adolesc Health. 2014;54(2):151-159.

[7] Markham CM, Tortolero SR, Peskin MF, et al. Sexual risk avoidance and sexual risk reduction interventions for middle school youth: A randomized controlled trial. J Adolesc Health. 2012;50(3):279-288.

[8] Tortolero SR, Markham CM, Peskin MF, Shegog R, Addy RC, Escobar-Chaves SL, Baumler ER. It's Your Game: Keep It Real: Delaying sexual behavior with an effective middle school program. J Adolesc Health. 2010;46(2):169-179. doi:10.1016/j.jadohealth.2009.06.008. PMID: 20113923. PMCID: PMC2818029.

[9] Rosen B, Meisman A, Hineline S. Using the Connect CCHMC Research Team. 2022.

[10] Welti K, Faccio B. Recommendations for developing survey items and outcome measures to evaluate teen pregnancy prevention programs. Child Trends. 2022.

[11] Coyle KK, Anderson PM, Franks HM, Glassman J, Walker JD, Charles VE. Romantic relationships: An important context for HIV/STI and pregnancy prevention programmes with young people. Sex Educ. 2014;14(5):582-596. doi:10.1080/14681811.2014.931278.

[12] Sullivan LF, Cheng MM, Harris KM, Brooks-Gunn J. I wanna hold your hand: The progression of social, romantic and sexual events in adolescent relationships. Perspect Sex Reprod Health. 2007;39(2):100-107. doi:10.1363/3910007. PMID: 17565623.

[13] Noll JG, Trickett PK, Putnam FW. A prospective investigation of the impact of childhood sexual abuse on the development of sexuality. J Consult Clin Psychol. 2003;71(3):575-586. doi:10.1037/0022-006X.71.3.575.

[14] Becnel J, Zeller MH, Noll JG, et al. Romantic, sexual, and sexual risk behaviours of adolescent females with severe obesity. Pediatr Obes. 2017;12(5):388-397. doi:10.1111/ijpo.12155.

[15] Centers for Disease Control and Prevention. Sexually transmitted diseases (STDs). U.S. Department of Health and Human Services. URL: <https://www.cdc.gov/std/default.htm>, (accessed 28 July 2026).

[16] Centers for Disease Control and Prevention. Reproductive health: Teen pregnancy. U.S. Department of Health and Human Services. URL: <https://www.cdc.gov/teenpregnancy/teens/index.htm>, (accessed 28 July 2026).

[17] Kirby D, Coyle K, Alton F, Rolleri L, Robin L. Reducing Adolescent Sexual Risk: A Theoretical Guide for Developing and Adapting Curriculum-Based Programs. ETR Associates. https://healtheducationresources.unesco.org/library/documents/reducing-adolescent-sexual-risk-theoretical-guide-developing-and-adapting, 2011 (accessed 28 July 2026).

[18] Norman CD, Skinner HA. eHEALS: The eHealth literacy scale. J Med Internet Res. 2006;8(4):e27. doi:10.2196/jmir.8.4.e27.

[19] Basen-Engquist K, Masse LC, Coyle K, Kirby D, Parcel GS, Banspach S, Nodora J. Validity of scales measuring the psychosocial determinants of HIV/STD-related risk behavior in adolescents. Health Educ Res. 1999;14(1):25-38. doi:10.1093/her/14.1.25.

[20] Bressani RV, Downs AC. Youth independent living assessment: Testing the equivalence of web and paper/pencil versions of the Ansell-Casey Life Skills Assessment. Comput Human Behav. 2002;18(4):453-464. doi:10.1016/S0747-5632(01)00053-X.

[21] Guttmacher Institute. National campaign to prevent teen and unplanned pregnancy questionnaire. Field Research Corporation. URL: <https://www.guttmacher.org/sites/default/files/dataset_documents/ns-questionnaire2009.pdf>, (accessed 28 July 2026).

[22] Whitehead R, Pringle J, Scott E, Milne D, McAteer J. The relationship between a trusted adult and adolescent health and education outcomes. Edinburgh, Scotland: NHS Health Scotland; 2019.

[24] Family & Youth Services Bureau. Participant Exit Survey Middle School Mathematica. URL: <https://www.prepeval.com/DataCollection/PREP_MS_Exit_Survey.pdf>, (accessed 28 July 2026).
